# Brain RAS: Hypertension and Beyond

**DOI:** 10.1155/2013/157180

**Published:** 2013-03-25

**Authors:** Marc de Gasparo, Robert C. Speth, Ovidiu C. Baltatu, Patrick Vanderheyden

**Affiliations:** ^1^Unit of Physiology, University of Jaén, 23071 Jaén, Spain; ^2^Department of Pharmaceutical Sciences, College of Pharmacy, Nova Southeastern University, Fort Lauderdale, FL 33328-2018, USA; ^3^Center of Biomedical Engineering, University Camilo Castelo Branco, 12247-004 São José dos Campos, SP, Brazil; ^4^Department of Molecular and Biochemical Pharmacology, Vrije Universiteit Brussel, 1050 Brussels, Belgium

Cardiac output and vascular resistance are the cornerstones of blood pressure regulation, which is achieved through neural, humoral, and local tissue factors.

The sympathetic nervous system (SNS) and the renin-angiotensin system (RAS) play a major role in the control of blood pressure. Angiotensins act as endocrine, paracrine, and autocrine regulators. The peripheral physiological and pathophysiological actions of the RAS are well established. The existence and functional relevance of the RAS in the brain is increasingly recognized as a major regulator of the cardiovascular (CV) system and a significant drug target for antihypertensive and other CV therapeutics. All the constituents of the RAS occur in the brain and participate in the regulation of blood pressure through sympathetic activation and vasopressin release. In addition, an interconnection between neurotransmitters and the brain RAS affects behavior and neurological diseases, for example, Parkinson's, and Alzheimer's diseases. Moreover, the clinical efficacy of renin and ACE inhibitors and angiotensin receptor blockers (ARBs) and the presence of their targets in the brain illustrate the synergistic interaction between brain and peripheral RAS. 

This special issue illustrates some aspects of the brain RAS pathway and function including its effect on the circadian rhythm of blood pressure.

The RAS has been described in the brain. Using subtype specific antibodies, C. Premer et al. observed selective expression of AT1a, AT1b, and AT2 receptor subtypes in neurons and glia in a large number of brain regions including the subfornical organ, median eminence, area postrema, paraventricular, and solitary tract nucleus of the rat brain as well as in the pituitary and adrenal. 

Ang II formation in the pineal gland and glial cells appears to depend on alternative pathways including chymase (L. A. Campos et al.). One possibility might be that the prorenin receptor (PRR) binds prorenin or renin from circulation to form Ang I and chymase to form Ang II. The brain PRR appears to initiate the brain angiotensin peptide formation (W. Li et al.). Indeed, PRR is expressed ubiquitously in the brain with the highest expression levels in the pituitary and frontal lobe. Recent findings indicate that PRR has RAS independent roles associated with the vacuolar proton-ATPase and the Wnt signaling pathways (W. Li et al.). PRR in the brain could play a pivotal role in neural regulation of blood pressure and body fluid homeostasis.

In addition, AT4/IRAP and Mas receptors are also present in the brain. Aminopeptidases (and other angiotensins degrading enzymes, e.g., ACE2 and endopeptidase), which form fragments such as Ang III, Ang IV, Ang 2–10, Ang 1–9, and Ang 1–7, are also the topic of several reports (A. B. Segarra et al.; M. A. Clark et al.). 

Formation of Ang III in the brain may promote hypertension while Ang IV, which inhibits vasopressinase activity and may have a therapeutic value for cognitive function in the brain.

There is still a debate regarding the relative importance of Ang II and Ang III in the brain. Using astrocytes in culture and an inhibitor of aminopeptidase A to prevent conversion of Ang II to Ang III, M. A. Clark et al. demonstrate that both Ang II and Ang III induce phosphorylation of MAPK and JNK and stimulate astrocyte growth equipotently.

Ang IV binds to the AT4 receptor. While the AT_4_ receptor has been convincingly shown to be the insulin-regulated aminopeptidase IRAP, (also known as vasopressinase and cysteine aminopeptidase), others have suggested that the physiological action of Ang IV may also be mediated through the tyrosine kinase c-Met receptor. Regardless of this controversy, binding of Ang IV causes inhibition of the catalytic activity of the peptidase activity of the IRAP receptor and therefore increases AVP and oxytocin, glucose uptake, and cognitive processes. Intracerebroventricular injection of Ang IV improves memory and learning in the rat. The potential of IRAP inhibitors able to cross the blood brain barrier is discussed by H. Andersson and M. Hallberg.

Clearly, the brain RAS regulates sympathetic activity and norepinephrine (NE) release (K. Tsuda), and hyperactivity of the SNS is clearly involved in the cardiovascular pathology. Ang II through the AT1 receptor and MAPK stimulation affects noradrenergic nerve terminals in the paraventricular nucleus of the hypothalamus (PVN), inhibiting K^+^ channel and stimulating Ca^++^ channels causing NE release. Also, brain aldosterone-mineralocorticoid receptor- (MR-) ouabain pathway might have a pivotal role in Ang II-induced neuronal activation and pressor responses (K. Tsuda). In contrast, Ang 1–7, a metabolite of both Ang I and Ang II, reduces NE release through BK and NO stimulation (M. Nautiyal et al.). 

Regulation of the baroreflex is central to CV regulation, and cardiac autonomic imbalance (decreased cardiovagal and increased sympathetic tone) causes baroreflex dysfunction. Ang II acting through the AT1 receptor and Ang 1–7 acting through the mas-receptor counterbalance each other in the brain through AMPK and mitochondrial NADPH oxidase stimulation and superoxide production (M. Nautiyal et al.; B. P. Campagnaro et al.). Ang II also stimulates dopamine *β*-hydroxylase in the striatum regulating the baroreflex (Tsuda). It also stimulates central cholinergic neuronal activity. The frontal cortex and CV system are reciprocally and asymmetrically organized: the frontal cortical activity appears to control CV function. Indeed, whereas there is an increased parasympathetic nervous system (PNS) activity in the left prefrontal cortex, one observes a decreased SNS activity in the right one (A. B. Segarra et al.). This may suggest that stimulation of the frontal cortical activity could lower blood pressure. 

Several contributions focus on the AT2 receptor, the functions of which still remain poorly understood. Interestingly, the hypothalamus expresses both AT1 and AT2 receptors that could counterbalance each other's effects (C. Premer et al.). Further, the AT2 receptor is involved in neuronal disorders and appears to play a major role in the regulation of cognitive function through a balance between phosphorylation and kinase activity versus phosphatase activity (M. O. Guimond and N. Gallo-Payet). AT2 receptors also stimulate neurite outgrowth, migration and excitability and reduce oxidative stress. Moreover, binding of Ang II to the AT2 receptor stimulates bradykinin and nitric oxide production and improves cerebral blood flow preventing ischemic damage and dementia (M. Mogi et al.). In addition, the AT2 receptor appears to play a role in metabolic syndrome as it regulates appetite and increases glucose uptake. Finally, it is proposed that the AT2 receptor behaves like a gate keeper of cellular and tissue homeostasis (M. O. Guimond and N. Gallo-Payet). 

Some signaling pathways involved in the AT2 receptor function are described. A family of AT2 receptor interacting proteins (ATIP) through the C-terminal domain of the receptor affect neurite outgrowth and neuronal differentiation (S. Rodrigues-Ferreira et al.). Such interaction of ATIP with the nicotinic acetylcholine receptor may also affect cognitive function (M. Mogi et al.). In fact, the evidence in favor of a neuroprotective action of AT2 receptor stimulation is compelling (M. Mogi et al.; M. O. Guimond and N. Gallo-Payet). 

Interestingly, the RAS in the brain and in the periphery is differently regulated by sodium (H. Takahashi). Whereas a sodium load suppresses the peripheral RAS and reduces sodium reabsorption from the renal tubules, it activates the brain RAS to retain more sodium. The mechanism involves the brain endothelial Na^+^ channel, activation of brain aldosterone, and endogenous digoxin-like substances, causing stimulation of the sympathetic system and increased blood pressure (H. Takahashi).

Circadian rhythms in neural and endocrine systems regulated by the molecular clock in the suprachiasmatic nucleus affect cardiovascular and metabolic disorders. Blood pressure variations during the day and night derive from the change in the dominant sympathetic tone. L. A. Campos et al. demonstrate here that Ang II acts upon AT1 receptors in the pineal gland to stimulate tryptophan hydroxylase activity and melatonin formation. This decreases sympathetic activity and increases parasympathetic activity. This reduces oxygen-free radicals and increases nitric oxide availability causing a decrease in blood pressure. Such behavior is typical of the physiological night tone reduction of blood pressure (dipper hypertensive patient), whereas the nondipper hypertensive shows an impaired melatonin formation during the night with an inverse circadian blood pressure profile. Pinealectomy reverses the condition, increasing sympathetic activity and the adrenocorticotropin axis. The brain RAS appears therefore to be a major regulator of circadian variation of blood pressure through its effect on light cycle shifts. Medical treatment coordinated with biological rhythms (chronotherapy) combined with inhibition of the brain RAS may be a means of individualizing treatment of hypertension related to circadian rhythms. Interestingly, increased melatonin release also improves insulin sensitivity. Supporting this finding, decreased melatonin is observed in type 2 diabetes and a melatonin gene receptor mutation was associated with increased risk of type 2 diabetes. Melatonin, a nutritional supplement, may therefore be a good therapeutic complement for diabetic patients. Although pineal Ang II stimulates melatonin (L. A. Campos et al.), a beneficial effect of this approach on type II diabetes remains to be demonstrated. 

S. Lattanzi et al. review the effectiveness of ARBs given in acute stroke. In a meta-analysis of three trials, they conclude that there is no place for blood pressure lowering treatment in the acute phase of stroke. However, there may be a U-shaped relationship between BP and outcome: too low blood pressure may result in brain hypoperfusion and thus in a worse outcome. Two ongoing large studies in acute ischemic and haemorrhagic stroke (ENOS and INTERACT) will help to select the best approach of blood pressure management.

 In summary, an abundant body of evidence indicates an important regulatory role for the brain RAS in cardiovascular homeostasis and disease beyond blood pressure regulation. Moreover, accumulating evidence reveals brain RAS roles in brain-specific functions and diseases such as cognitive dysfunction, dementia, Alzheimer's and Parkinson's diseases. Further research is needed to better understand detailed mechanisms of brain RAS in these and other diseases to possibly develop new diagnostic strategies.

*Marc de Gasparo* *Marc de Gasparo* *Robert C. Speth* *Robert C. Speth* *Ovidiu C. Baltatu* *Ovidiu C. Baltatu* *Patrick Vanderheyden* *Patrick Vanderheyden*

